# Association between the weight-adjusted-waist index and abdominal aortic calcification in United States adults: Results from the national health and nutrition examination survey 2013–2014

**DOI:** 10.3389/fcvm.2022.948194

**Published:** 2022-09-14

**Authors:** Feng Xie, Yuan Xiao, Xiaozhong Li, Yanqing Wu

**Affiliations:** ^1^Department of Cardiovascular Medicine, The Second Affiliated Hospital of Nanchang University, Nanchang, Jiangxi, China; ^2^Department of Ultrasonic, Jiangxi Pingxiang People’s Hospital, Pingxiang, Jiangxi, China

**Keywords:** weight-adjusted-waist index, abdominal aortic calcification, NHANES, cross-sectional study, cardiovascular disease

## Abstract

**Background:**

Abdominal aortic calcification (AAC) is recognized as a strong predictor of cardiovascular disease (CVD) events. This study aimed to evaluate the association between weight-adjusted-waist index (WWI) and AAC in United States adults aged ≥ 40 years.

**Materials and methods:**

Data were derived from the 2013–2014 National Health and Nutrition Examination Survey (NHANES). WWI was calculated as waist circumference divided by the square root of weight. AAC scores were quantified by the Kauppila scoring system, and severe AAC was defined as an AAC score ≥ 6. Weighted multivariable regression analysis and subgroup analysis were performed to evaluate the relationship between WWI with AAC scores and severe AAC. The restricted cubic spline model was used for the dose-response analysis.

**Results:**

A total of 2,772 participants were included with the mean WWI of 11.17 ± 0.73 cm/√kg and mean AAC score of 1.48 ± 3.27. The prevalence of severe AAC was 9.64%. WWI was positively associated with higher AAC scores [β = 0.95, 95% confidence interval (CI): 0.65–1.25, *P* < 0.001] and increased risk of severe AAC [odds ratio (OR) = 1.82; 95% CI: 1.20–2.75; *P* = 0.005]. A nearly linear relationship between the WWI and the odds of severe AAC was found after adjustment for multiple potential covariates (*P* for non-linear = 0.625). Subgroup analysis indicated that the association between WWI and AAC was similar in different population settings.

**Conclusion:**

Higher WWI was associated with higher AAC score and increased risk of severe AAC in United States adults. Further studies are needed to confirm this relationship.

## Introduction

Cardiovascular disease (CVD) is the largest single contributor to global mortality, with the number of deaths steadily increasing from 12.1 million in 1990 to 18.6 million in 2019 (1). Atherosclerosis is the major underlying cause of CVD, which substantially elevates the incidence and risk of CVD (2). Hence, it is crucial to accurately detect the presence of atherosclerosis in the subclinical stages for the prevention and intervention of CVD.

As a marker of subclinical atherosclerosis, abdominal aortic calcification (AAC) is recognized as a strong predictor of CVD events (3). Previous studies have demonstrated that AAC measured on spine X-rays was independently associated with myocardial infarction and stroke (4, 5). Moreover, the Multi-Ethnic Study found that the predictive value of AAC for CVD was independent of coronary artery calcification (CAC) and that AAC was more strongly associated with total mortality than CAC (6). Therefore, AAC has attracted increasing attention in recent years.

Obesity is an increasingly severe public health problem. The prevalence of obesity has increased dramatically in the United States (US) and around the world over recent decades (7). By 2025, the global prevalence of obesity will reach 18% in men and surpass 21% in women (8). Moreover, cumulative evidence has established that obesity is associated with an increased prevalence of hypertension, diabetes mellitus, and other CVDs (9–11). Despite that, traditional obesity-related indicators, such as body mass index (BMI) and waist circumference (WC), do not differentiate between muscle mass and fat mass (12). Therefore, these measures do not accurately reflect the association between obesity and adverse health outcomes (13).

In 2018, Park et al. proposed a new anthropometric index, the weight-adjusted-waist index (WWI), which was positively correlated with fat mass but negatively correlated with muscle mass (14, 15). Moreover, several studies have demonstrated that WWI is associated with an elevated risk of incident hypertension and cardiovascular mortality (16, 17). However, to our knowledge, no previous study has investigated the relationship between WWI and AAC.

Taken together, it is important to evaluate the relationship between WWI and AAC in regular civilians in order to prevent the incidence and improve the prognosis of CVD more effectively. Accordingly, the objective of this study was to explore the association between WWI and AAC in United States adults based on data obtained from the National Health and Nutrition Examination Survey (NHANES). We hypothesized that WWI was positively associated with AAC score and the risk of severe AAC.

## Materials and methods

### Study population and design

This cross-sectional study used data from NHANES, a program of studies designed to assess the health and nutritional status of the United States population using a multistage and stratified probability design. The details of the NHANES study design and protocols have been previously described (18). NHANES is conducted by the National Center for Health Statistics (NCHS), which is part of the Centers for Disease Control and Prevention (CDC) and has the responsibility for producing vital and health statistics for the nation. All procedures were approved by the NCHS Research Ethics Review Board and written informed consent was obtained from all participants.

**FIGURE 1:**
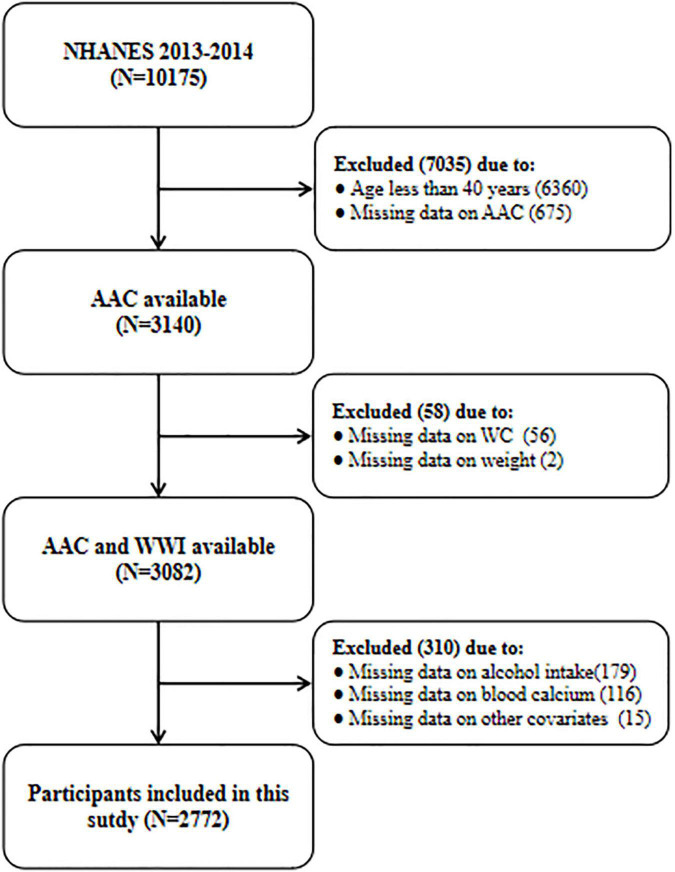
The flow chart of participant selection. NHANES, National Health and Nutrition Examination Survey; AAC, abdominal aortic calcification; WWI, weight-adjusted-waist index; WC, waist circumference.

A total of 10,175 participants were enrolled in the 2013–2014 NHANES cycle, but only people ≥ 40 years old underwent dual-energy X-ray absorptiometry (DXA) to calculate the AAC score (*N* = 3,815). Then, we further excluded participants with missing data on AAC (*N* = 675), WC (*N* = 56), weight (*N* = 2), and other covariates (*N* = 310). Finally, 2,772 participants with complete information were included in the current study. All data used in this manuscript have been made publicly available at the NCHS website.^1^ The detailed flow chart about participant selection was shown in Figure 1.

### Data collection and measurements

The data were derived from home interviews and mobile examination center (MEC) questionnaires administered by trained staff. The demographic and lifestyle variables included age, gender, race/ethnicity, educational level, and alcohol drinking status. Race/ethnicity was classified as Mexican American, other Hispanic, non-Hispanic black, non-Hispanic white, and other races. We further combined Mexican American and other Hispanic into one group (Hispanic group), and non-Hispanic black, non-Hispanic white and other races into another (non-Hispanic group) in the subgroup analysis (Figure 3). Educational level was categorized as less than 9th grade, 9–11th grade, high school graduate, some college or AA degree, and college graduate or above. Alcohol drinking status was evaluated by “Had at least 12 alcohol drinks per year.” Anthropometric information, including weight, height, and WC. BMI was calculated as weight in kilograms divided by height in meters squared (kg/m^2^). The health conditions, including hypertension (HTN), diabetes mellitus (DM), and coronary heart disease (CHD), were collected based on the self-report of participants.

**FIGURE 2:**
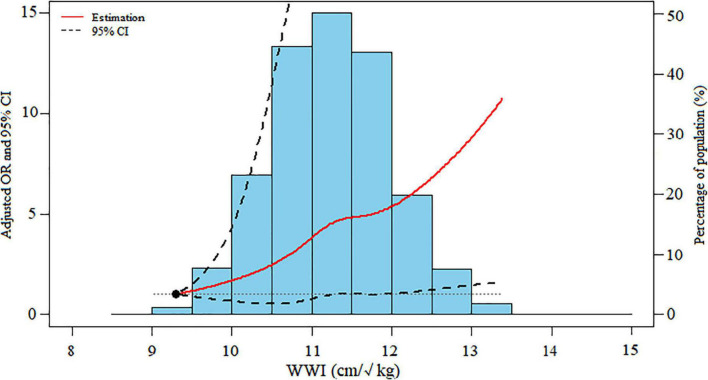
Dose-response association between WWI levels and severe AAC. Data were ORs (solid line) and 95% CIs (dashed lines) from multiple logistic regression analysis with restricted cubic splines, with WWI 9.30 cm/√kg as the reference. WWI, weight-adjusted-waist index; AAC, abdominal aortic calcification; OR, odds ratio; CI, confidence interval.

**FIGURE 3:**
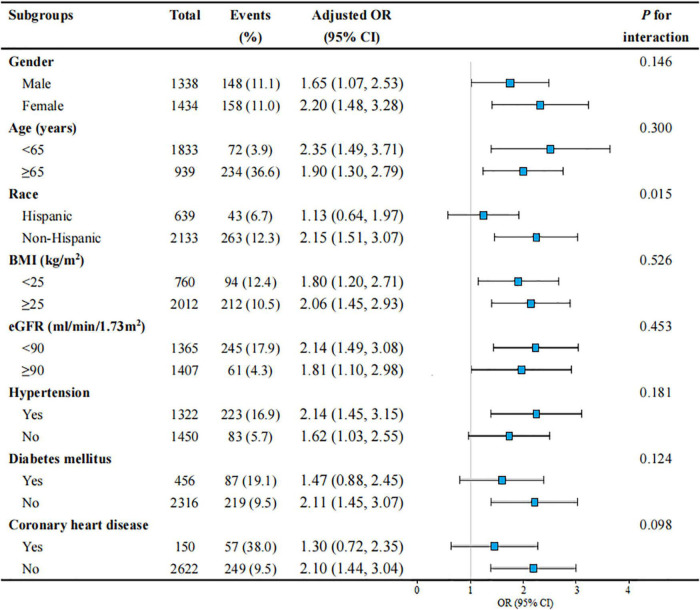
Subgroup analyses of the association between WWI level and severe AAC in United States adults from NHANES 2013–2014. All presented covariates were adjusted (as Model 3) except the corresponding stratification variable. WWI, weight-adjusted-waist index; AAC, abdominal aortic calcification; US, the United States; NHANES, National Health and Nutrition Examination Survey; OR, odds ratio; CI, confidence interval; BMI, body mass index; eGFR, estimated glomerular filtration rate.

The Beckman UniCel DxC800 Synchron System (Beckman, Fullerton, CA, United States) used a timed-endpoint method to measure serum total cholesterol, triglycerides, and uric acid; a timed-rate method to measure serum phosphorus; an indirect (or diluted) I.S.E. (ion selective electrode) methodology to measure serum calcium; the Jaffe rate method (kinetic alkaline picrate) to measure serum creatinine; a bichromatic digital endpoint method to measure albumin; and a timed-endpoint Diazo method (Jendrassik-Grof) to measure serum total bilirubin. Total 25-hydroxyvitamin D was measured by a standardized liquid chromatography-tandem mass spectrometry method and hemoglobin A1c was measured by high-performance liquid chromatography. Besides, estimated glomerular filtration rate (eGFR) was calculated using the Chronic Kidney Disease Epidemiology Collaboration (CKD-EPI) creatinine equation (19).

### Exposure variable and outcomes

In the current study, WWI (cm/√kg) was designed as an exposure variable, which was calculated as WC (cm) divided by the square root of weight (kg) (14). The anthropometry examinations were performed in the MEC by trained health technicians and monitored through direct observation, data reviews, and periodic expert examiner evaluations. The full procedure, including the protocols and quality control, was described at https://wwwn.cdc.gov/nchs/nhanes/ContinuousNhanes/Manuals.aspx?BeginYear=2013. Weight was measured to the nearest 0.1 kg using a digital weight scale. Each subject wore a standard MEC examination gown before weighting, then stood in the center of the digital scale with hands at sides and eyes looking straight forward. WC was measured using a retractable steel measuring tape. The iliac crests were palpated bilaterally and a horizontal line was drawn just above the uppermost lateral border of the right ilium. Then, the right midaxillary line was drawn. At the point where the two lines crossed, the measuring tape was positioned in the horizontal plane. Take the measurement to the nearest 0.1 cm at the end of the individual’s normal expiration.

The AAC score and severe AAC were designed as outcome variables. AAC was obtained from a lateral scan of the lumbar spine (vertebrae L1–L4) using DXA. The Kauppila score system was applied to quantify the AAC score. DXA scans were performed on eligible survey participants aged ≥ 40 years. Meeting any of the following criteria will be excluded: (1) self-report pregnancy and/or positive urine pregnancy test; (2) self-reported history of radiographic contrast material (barium) use in past 7°days; (3) self-reported weight over 450 pounds; (4) self-reported history of scoliosis with surgical rod implantation. A Kauppila score ≥ 6 was defined as severe AAC, which was recognized as severe vascular calcification according to previous studies (20, 21).

### Statistical analysis

According to NHANES analytic guidelines, data were presented as weighted mean ± standard deviation (SD) for continuous variables and frequency (weighted percentages) for categorical variables. WWI was analyzed as a continuous as well as a categorical variable (quintiles), with the first quartile as the reference. The differences in baseline characteristics by WWI quintiles were compared using one-way analysis of variance (ANOVA) tests for continuous variables and Chi-square test for categorical variables. Multivariable linear regressions were used to evaluate the relationship between WWI and AAC score. The odds ratios (ORs) and 95% confidence intervals (CIs) for severe AAC with WWI were determined using multivariate logistic regression models. Potential covariates including age, gender, race, BMI, WC, education level, alcohol drinking status, eGFR, hemoglobin A1c, serum creatinine, serum uric acid, total cholesterol, triglycerides, total bilirubin, albumin, total 25-hydroxyvitamin D, calcium, phosphorus, HTN, DM, and CHD. The restricted cubic spline model was used for the dose-response analysis. Furthermore, subgroup analysis stratified by gender (female or male), age (< 65 or ≥ 65 years), race (Hispanic or non-Hispanic), BMI (< 25 or ≥ 25 kg/m^2^), eGFR (< 90 or ≥ 90 ml/min/1.73°m^2^), HTN (yes or no), DM (yes or no) and CHD (yes or no) was conducted by stratified multivariate regression analysis. A receiver operating characteristic (ROC) curve was used to analyze the predictive value of the different indices for severe AAC.

All analyses were performed using R version 4.0.3 (www.R-project.org) and EmpowerStates (www.empowerstats.com). A two-sided *P* value of < 0.05 was considered statistically significant.

## Results

### Baseline characteristics

The weighted baseline characteristics of all participants are shown in Table 1. A total of 2,772 participants (1,338 men and 1,434 women) with a mean age of 57.72 ± 11.48 years were included in this study. The mean AAC score was 1.48 ± 3.27 overall, and 0.50 ± 1.44, 1.16 ± 2.70, 1.57 ± 3.19, 1.94 ± 3.74, and 2.53 ± 4.54 for Quintiles 1, 2, 3, 4, and 5, respectively. The mean WWI was 11.17 ± 0.73 cm/√kg overall, and the ranges of WWI quintiles 1–5 were 8.79–10.60, 10.61–11.02, 11.03–11.43, 11.44–11.85, and 11.86–14.79 cm/√kg, respectively. Participants with higher WWI levels were older and more likely to be female; to have higher AAC score, BMI, WC, hemoglobin A1c, triglycerides, and serum uric acid; to have a higher rate of HTN, DM, and CHD; to have a lower rate of alcohol drinking status; and to have lower total bilirubin and albumin levels (all *P* < 0.01).

**TABLE 1:** Baseline characteristics of all participants stratified by quintiles of weight-adjusted-waist index (WWI).

Variables^#^	Total	Quintile categories of WWI (cm/√kg)					*P-*value
		Quintile 1 (8.79–10.60)	Quintile 2 (10.61–11.02)	Quintile 3 (11.03–11.43)	Quintile 4 (11.44–11.85)	Quintile 5 (11.86–14.79)	
Participants	2772	555	554	554	554	555	
AAC score	1.48 ± 3.27	0.50 ± 1.44	1.16 ± 2.70	1.57 ± 3.19	1.94 ± 3.74	2.53 ± 4.54	< 0.001
WWI (cm/√kg)	11.17 ± 0.73	10.23 ± 0.32	10.83 ± 0.12	11.22 ± 0.12	11.63 ± 0.12	12.27 ± 0.38	< 0.001
Age (years)	57.72 ± 11.48	51.71 ± 8.99	55.98 ± 10.61	57.63 ± 11.25	61.35 ± 11.39	64.03 ± 11.23	< 0.001
Female (%)	1434 (51.71)	222 (44.47)	243 (45.82)	274 (48.17)	305 (53.88)	390 (70.09)	< 0.001
Race (%)							< 0.001
Mexican American	368 (6.84)	36 (3.33)	66 (6.89)	72 (6.31)	99 (9.98)	95 (8.79)	
Other hispanic	271 (4.67)	32 (3.07)	56 (5.28)	63 (4.74)	60 (5.63)	60 (4.98)	
Non-hispanic white	1254 (72.19)	237 (72.37)	231 (69.86)	250 (74.25)	256 (70.79)	280 (73.68)	
Non-hispanic black	525 (9.58)	163 (14.08)	109 (9.36)	104 (8.41)	82 (7.99)	67 (6.99)	
Other race	354 (6.72)	87 (7.15)	92 (8.61)	65 (6.29)	57 (5.61)	53 (5.56)	
Educational level (%)							< 0.001
Less than 9th grade	240 (4.61)	22 (1.84)	32 (3.82)	43 (4.69)	59 (6.09)	84 (7.57)	
9–11th grade	366 (9.94)	50 (5.34)	68 (9.45)	83 (10.07)	77 (10.36)	88 (15.98)	
High school graduate	629 (21.89)	112 (18.53)	122 (19.79)	137 (24.89)	116 (20.97)	142 (26.04)	
Some college or AA degree	790 (29.94)	154 (24.82)	149 (27.51)	149 (30.99)	184 (37.00)	154 (31.02)	
College graduate or above	746 (33.61)	217 (49.47)	183 (39.43)	142 (29.35)	118 (25.57)	86 (19.36)	
BMI (kg/m^2^)	28.55 ± 5.45	25.57 ± 4.55	27.35 ± 4.62	28.86 ± 4.86	30.28 ± 5.24	31.73 ± 5.86	< 0.001
WC (cm)	99.96 ± 13.51	88.96 ± 9.97	95.99 ± 10.67	101.27 ± 11.07	106.10 ± 11.55	111.16 ± 12.35	< 0.001
Alcohol drinking status (%)	1984 (78.27)	445 (85.90)	417 (81.77)	404 (80.46)	379 (74.58)	339 (65.31)	< 0.001
eGFR (ml/min/1.73m^2^)	98.10 ± 34.38	95.93 ± 25.98	99.12 ± 30.25	101.26 ± 35.49	99.31 ± 36.82	94.65 ± 43.10	0.009
Hemoglobin A1c (%)	5.77 ± 1.01	5.42 ± 0.52	5.65 ± 0.94	5.73 ± 0.96	5.97 ± 1.12	6.19 ± 1.28	< 0.001
Total bilirubin (umol/L)	11.15 ± 5.16	11.94 ± 4.47	11.28 ± 4.72	11.29 ± 5.02	11.17 ± 6.83	9.77 ± 4.35	< 0.001
Albumin (g/L)	42.53 ± 3.00	43.27 ± 2.80	42.79 ± 2.95	42.69 ± 3.02	42.07 ± 2.99	41.52 ± 2.99	< 0.001
Total cholesterol (mmol/L)	5.08 ± 1.11	5.09 ± 0.94	5.12 ± 1.11	5.04 ± 1.10	5.09 ± 1.31	5.05 ± 1.10	0.754
Triglycerides (mmol/L)	1.80 ± 1.71	1.37 ± 1.01	1.72 ± 1.19	1.77 ± 1.06	2.16 ± 3.12	2.15 ± 1.41	< 0.001
Serum creatinine (umol/L)	81.95 ± 37.15	82.58 ± 19.45	80.97 ± 32.81	82.58 ± 53.17	82.61 ± 43.25	80.85 ± 28.23	0.857
Serum uric acid (umol/L)	321.22 ± 81.34	305.28 ± 81.39	317.12 ± 80.41	322.64 ± 73.86	332.69 ± 84.31	333.48 ± 83.94	< 0.001
Serum total calcium (mmol/L)	2.36 ± 0.09	2.36 ± 0.09	2.35 ± 0.09	2.37 ± 0.09	2.36 ± 0.08	2.37 ± 0.09	0.008
Serum phosphorus (mmol/L)	1.23 ± 0.18	1.22 ± 0.18	1.23 ± 0.17	1.21 ± 0.18	1.22 ± 0.19	1.24 ± 0.18	0.079
Total 25-hydroxyvitamin D (nmol/L)	75.39 ± 29.39	77.45 ± 28.59	73.90 ± 27.47	76.64 ± 28.32	73.86 ± 29.37	74.49 ± 33.39	0.115
Hypertension (%)	1322 (43.87)	161 (25.89)	238 (40.27)	274 (44.25)	292 (52.11)	357 (62.77)	< 0.001
Diabetes mellitus (%)	456 (12.97)	31 (4.50)	54 (7.86)	86 (12.74)	119 (17.06)	166 (26.21)	< 0.001
Coronary heart disease (%)	150 (4.92)	13 (1.25)	24 (4.73)	25 (3.56)	38 (6.90)	50 (9.58)	< 0.001

^#^Values are presented as weighted mean ± standard deviation or frequency (weighted percentages) when appropriate.

AAC, abdominal aortic calcification; WWI, weight-adjusted-waist index; BMI, body mass index; WC, waist circumference; eGFR, estimated glomerular filtration rate.

### Association of weight-adjusted-waist index with abdominal aortic calcification score

As shown in Table 2, WWI was positively associated with AAC scores (Crude model: β = 0.96, 95% CI: 0.79–1.12, *P* < 0.001; Model 1: β = 0.32, 95% CI: 0.15–0.49, *P* < 0.001; Model 2: β = 1.02, 95% CI: 0.74–1.29, *P* < 0.001). In the fully adjusted model (Model 3), this positive association was still stable (β = 0.95, 95% CI: 0.65–1.25, *P* < 0.001), indicating that each unit of increased WWI was associated with 0.95 higher unit of AAC score. We also converted WWI from a continuous variable to a categorical variable (quintiles) for sensitivity analysis. AAC score increased with the higher WWI quintile group. The average AAC scores of Quintiles 2, 3, 4, and 5 were 0.46, 0.94, 1.13, and 1.65 units higher compared with the lowest quintile (Quintile 1), respectively. However, BMI was negatively correlated with AAC scores (Model 2: β = −0.06, 95% CI: −0.11 to −0.01; *P* < 0.05) (Supplementary Table 1 in Supplementary Information), and WC (/10cm) was not correlated with AAC scores (Model 2: β = −0.02, 95% CI: −0.24 to 0.19; *P* > 0.05) (Supplementary Table 2 in Supplementary Information). In addition, compared with BMI and WC, WWI was also significantly correlated with the AAC score with Pearson’s correlation coefficient *r* = 0.213 (*P* < 0.001) (Supplementary Table 3 in Supplementary Information).

**TABLE 2:** Association between weight-adjusted-waist index (WWI) level and abdominal aortic calcification (AAC) score in United States adults from national health and nutrition examination survey (NHANES) 2013–2014.

WWI (cm/√kg)	Crude model β (95% CI)	Model 1 β (95% CI)	Model 2 β (95% CI)	Model 3 β (95% CI)
Per 1°cm/√kg increase	0.96 (0.79, 1.12)	0.32 (0.15, 0.49)	1.02 (0.74, 1.29)	0.95 (0.65, 1.25)
Categorical				
Quintile 1	Reference	Reference	Reference	Reference
Quintile 2	0.66 (0.29, 1.02)	0.19 (−0.15, 0.53)	0.54 (0.18, 0.90)	0.46 (0.10, 0.82)
Quintile 3	1.07 (0.71, 1.43)	0.43 (0.09, 0.77)	1.01 (0.61, 1.40)	0.94 (0.54, 1.33)
Quintile 4	1.44 (1.06, 1.82)	0.42 (0.05, 0.78)	1.25 (0.78, 1.72)	1.13 (0.65, 1.60)
Quintile 5	2.03 (1.65, 2.41)	0.72 (0.34, 1.11)	1.85 (1.29, 2.40)	1.65 (1.08, 2.23)
*P* for trend	< 0.001	0.002	< 0.001	< 0.001

Model 1: adjusted for age, gender and race.

Model 2: adjusted for age, gender, race, BMI, and WC.

Model 3: adjusted for model 2 + education level, alcohol drinking status, eGFR, hemoglobin A1c, serum creatinine, serum uric acid, total cholesterol, triglycerides, total bilirubin, albumin, total 25-hydroxyvitamin D, calcium, phosphorus, hypertension, coronary heart disease, and diabetes mellitus.

WWI, weight-adjusted-waist index; AAC, abdominal aortic calcification; US, the United States; NHANES, National Health and Nutrition Examination Survey; β, effect size; CI, confidence interval.

### Association of weight-adjusted-waist index with severe abdominal aortic calcification

Table 3 shows the weighted prevalence of severe AAC and the association between severe AAC and the WWI as continuous and categorical variables. The weighted prevalence of severe AAC was 9.64% (306 cases). When WWI was analyzed as a continuous variable, we found that increased WWI was associated with a higher risk of severe AAC (Crude model: OR = 2.34; 95% CI: 1.97–2.79; *P* < 0.001; Model 1: OR = 1.33; 95% CI: 1.08–1.64; *P* = 0.007; Model 2: OR = 2.11; 95% CI: 1.49–2.98; *P* < 0.001). In Model 3 which adjusted for all covariates, the results indicated that each unit of increased WWI was associated with 82% increased risk of severe AAC (OR = 1.82; 95% CI: 1.20–2.75; *P* = 0.005). In sensitivity analysis, the adjusted OR (reference to Quartile 1) was 2.54 (95% CI: 1.17–5.54; *P* = 0.019) for Quartile 2, 4.58 (95% CI: 2.09–10.08; *P* < 0.001) for Quartile 3, 4.10 (95% CI: 1.75–9.58; *P* = 0.001) for Quintile 4, and 5.75 (95% CI: 2.27–14.55; *P* < 0.001) for Quartile 5, indicating a stable positive association between higher WWI and increased risk of severe AAC (*P* for trend < 0.001). Nevertheless, there was no association between BMI (Model 2: OR = 0.95, 95% CI: 0.89–1.03; (Supplementary Table 4 in Supplementary Information) or WC (Model 2: OR = 1.00, 95% CI: 0.97–1.03; (Supplementary Table 5 in Supplementary Information) with severe AAC.

**TABLE 3:** Association between weight-adjusted-waist index (WWI) level and severe abdominal aortic calcification (AAC) in United States adults from national health and nutrition examination survey (NHANES) 2013–2014.

WWI (cm/√kg)	Severe AAC (n,%)	Crude model OR (95% CI)	Model 1 OR (95% CI)	Model 2 OR (95% CI)	Model 3 OR (95% CI)
Per 1°cm/√kg increase	306 (9.64)	2.34 (1.97, 2.79)	1.33 (1.08, 1.64)	2.11 (1.49, 2.98)	1.82 (1.20, 2.75)
Categorical					
Quintile 1	17 (1.95)	Reference	Reference	Reference	Reference
Quintile 2	44 (6.80)	3.67 (1.77, 7.60)	2.17 (1.02, 4.59)	2.91 (1.35, 6.29)	2.54 (1.17, 5.54)
Quintile 3	64 (11.04)	6.24 (3.13, 12.42)	3.19 (1.56, 6.54)	4.99 (2.32, 10.72)	4.58 (2.09, 10.08)
Quintile 4	76 (12.85)	7.41 (3.78, 14.53)	2.61 (1.29, 5.28)	5.06 (2.26, 11.32)	4.10 (1.75, 9.58)
Quintile 5	105 (18.10)	11.11 (5.76, 21.44)	3.19 (1.62, 6.28)	7.82 (3.35, 18.25)	5.75 (2.27, 14.55)
*P* for trend		< 0.001	0.007	< 0.001	< 0.001

Model 1: adjusted for age, gender, and race;

Model 2: adjusted for age, gender, race, BMI, and WC;

Model 3: adjusted for model 2 + education level, alcohol drinking status, eGFR, hemoglobin A1c, serum creatinine, serum uric acid, total cholesterol, triglycerides, total bilirubin, albumin, total 25-hydroxyvitamin D, calcium, phosphorus, hypertension, coronary heart disease, and diabetes mellitus.

WWI, weight-adjusted-waist index; AAC, abdominal aortic calcification; US, the United States; NHANES, National Health and Nutrition Examination Survey; OR, odds ratio; CI, confidence interval.

The dose-response analysis with a restricted cubic spline model shows a nearly linear relationship between the WWI and the odds of severe AAC after adjustment for multiple potential covariates in Model 3 (*P* for non-linear = 0.625) (Figure 2).

### Subgroup analysis

Subgroup analyses were performed to examine whether the association between WWI and severe AAC was stable among different population settings (Figure 3). Besides, an interaction test of gender, age, race, BMI, eGFR, HTN, DM, and CHD was also conducted. The effect of WWI on AAC was more significant in the non-Hispanic subgroup (non-Hispanic: OR = 2.15, 95% CI: 1.51–3.07; Hispanic: OR = 1.13, 95% CI: 0.64–1.97; *P* for interaction = 0.015). WWI was also significantly associated with an increased risk of severe AAC in the non-DM subgroup (OR = 2.11, 95% CI: 1.45–3.07) and non-CHD subgroup (OR = 2.10, 95% CI: 1.44–3.04), but this positive association did not meet the statistical significance in their counterparts, with a *P* for interaction of 0.124 and 0.098, respectively. In addition, the positive association between WWI and AAC was consistent in the following subgroups: gender, age, BMI, eGFR, and HTN (all *P* for interaction > 0.05).

### Receiver operating characteristic curves of each obesity indices

The ROC curves of the different indices for severe AAC are shown in Supplementary Figure 1 (Supplementary Information). According to the ROC analyses, WWI had the highest area under the curve (AUC) of 0.651 (95% CI: 0.620–0.681), followed by WC (AUC: 0.487) and BMI (AUC: 0.429).

## Discussion

To our knowledge, this cross-sectional study was the first to demonstrate the relationship between WWI and AAC among United States non-institutionalized civilians. The results indicated that higher WWI was independently associated with higher AAC score and increased risk of severe AAC after adjusting for multiple potential covariates including demographics, laboratory measurements, comorbidities, and others, presenting a nearly linear dose-response relationship. Further stratified analyses suggested that WWI was positively associated with severe AAC occurrence in different population settings. Moreover, our findings showed that WWI was superior to BMI and WC in predicting severe AAC.

With the increasing prevalence of obesity and obesity-related disorders worldwide, it is critical to accurately assess obesity and then to identify individuals at risk of metabolic and cardiovascular diseases in clinical practice. However, most obesity-related anthropometric indices such as BMI and WC have inherent limitations as they do not distinguish between muscle mass and fat mass (13, 22). This may contribute to the seemingly paradoxical relationship between obesity and some health outcomes (13, 23). Notably, WWI is a newly-developed parameter of obesity that more accurately estimates whole-body fat percentage compared to traditional equations based on BMI or WC (14, 15). Previously, in the Korean National Health Insurance Cohort study including 465,629 participants, Park et al. reported that WWI was positively associated with cardiometabolic morbidity and cardiovascular mortality, which was not shown in BMI or WC (14). Similarly, Ding et al. found a non-linear positive association of WWI levels with the risk of all-cause and cardiovascular mortality in the China Hypertension Survey, independent of BMI and WC (17). Furthermore, compared with the lowest WWI category (< 9.94 cm/√kg), the Rural Chinese Cohort Study demonstrated that the highest WWI category (≥ 10.91 cm/√kg) was associated with 50% increased risk of incident hypertension (OR: 1.50, 95% CI: 1.24–1.82) (16). These findings suggest that WWI may be a comprehensive and superior indicator of obesity and could be used to predict obesity-related disorders.

In our analysis, higher WWI, but not BMI or WC, was independently associated with higher AAC score and increased odds of severe AAC, indicating that WWI was strongly associated with adverse health outcomes. WWI was developed based on the formula [In (WC) = β_0_ + β_1_ In (weight) + ε], which standardizes WC for body weight by the least squared regression of the logarithm-transformed WC on the logarithm-transformed weight (14). Therefore, WWI may combine the advantages of WC but weaken the correlation with BMI, enabling it to assess both fat and muscle mass components, regardless of the BMI category (15). Nevertheless, BMI does not differentiate between muscle mass and fat mass (13). Moreover, its assessment of body fat in older adults is not as accurate as that in younger adults (12). This was consistent with our data that the average age of participants was nearly 60 years old (57.72 ± 11.48 years). This may contribute to the “obesity paradox” between BMI and AAC scores in our study.

The mechanism underlying the positive association between WWI and AAC may be correlated with metabolic abnormalities. As mentioned above, WWI was positively correlated with abdominal fat area and visceral fat area but negatively correlated with abdominal muscle area, suggesting that the increase in WWI may reflect a state of excessive body fat accumulation (15, 24). Indeed, several studies have confirmed that visceral obesity was independently associated with AAC (OR: 6.63, 95% CI: 1.90–23.14), while lower visceral fat density was associated with a lower risk of AAC (OR: 0.79, 95% CI: 0.67–0.92), which is in agreement with our findings (25, 26). Moreover, Abolnezhadian et al. uncovered that the WWI levels were significantly higher in metabolically unhealthy groups than in metabolically healthy groups, even with a similar degree of obesity (27). Accordingly, in our study, higher WWI levels were also associated with increased levels of hemoglobin A1c, triglycerides, and serum uric acid, as well as a higher prevalence of HTN, DM, and CHD, which were considered the makers or products of metabolic abnormalities. It has been established that factors such as hemoglobin A1c, serum uric acid, and hypertension are closely associated with vascular calcification (28–30). Additionally, Yang et al. also reported a significant association between metabolic syndrome and AAC in the United States adult population, supporting our current results (31). Another possible mechanism of the WWI-AAC association is obesity-related inflammation. The increased WWI may reflect a state of adipose tissue dysfunction, which produces a variety of proinflammatory cytokines and adipocytokines such as interleukin-6 (IL-6), tumor necrosis factor-alpha (TNF-α), and resistin, leading to inflammatory response, endothelial dysfunction, insulin resistance (IR), and ultimately atherosclerosis and arterial calcification (32–34). Besides, aging may partially explain the WWI-AAC association, as it is well-established that arterial stiffness and vascular calcification increase with physiological aging (35, 36). And findings from our study, along with a previous report, show that WWI was significantly higher with increasing age (24).

Stratified analyses demonstrated that there was no dependence of gender, age, BMI, eGFR, HTN, DM, and CHD on the positive association between WWI and severe AAC (all *P* for interaction > 0.05), indicating that these positive correlations were similar in different population settings. Although the OR in the Hispanic subgroup was significantly lower than that of the corresponding subgroup, this may be related to the small sample size of the extensive AAC (43 participants) after stratification. Since the limited sample size may lead to potential bias, validation in larger specific populations is needed in the future.

Our study was based on NHANES data, a nationwide population-based sampling survey with rigorous study protocols and quality controls. Moreover, we adjusted for multiple potential covariates to ensure that our results were reliable. Due to the simple calculation and lack of radiation exposure, WWI could be used as a practical tool for the management and intervention of vascular calcification in clinical practice. Furthermore, since AAC was considered a strong predictor of CVD, it is possible that WWI may become a superior anthropometric index to BMI or WC in predicting CVD risk in overweight or obese individuals in medical and health institutions. Despite that, this study has several limitations. Firstly, the causal relationship between WWI and AAC cannot be determined due to the cross-sectional nature of this study. Secondly, although we adjusted for many important covariates, we could not completely rule out the influence of other possible confounding factors. Thirdly, the AAC score was measured only in people aged 40 years and older, which may affect the external validity.

## Conclusion

Our study demonstrated that higher WWI was independently associated with higher AAC score and increased risk of severe AAC in a representative sample of United States adults. The results provide a basis for future research on the relationship between WWI and CVD incidence and prognosis.

## Data availability statement

The datasets presented in this study can be found in online repositories. The names of the repository/repositories and accession number(s) can be found below: https://www.cdc.gov/nchs/nhanes/index.htm.

## Ethics statement

The studies involving human participants were reviewed and approved by the NCHS Ethics Review Board. The patients/participants provided their written informed consent to participate in this study.

## Author contributions

YW designed this study and revised the manuscript. FX and YX contributed equally to the writing of this manuscript. XL critically edited the manuscript for important intellectual content. All the authors approved the final version.

## Footnotes

1
https://wwwn.cdc.gov/nchs/nhanes/continuousnhanes/default.aspx?BeginYear=2013
