# Supplementary material for: Association between the weight-adjusted-waist index and abdominal aortic calcification in United States adults: Results from the national health and nutrition examination survey 2013–2014
Source: Front Cardiovasc Med. 2022 Sep 14;9:948194. doi: 10.3389/fcvm.2022.948194 (PMC9515490; doi:10.3389/fcvm.2022.948194)
Supplement: Supplementary file 1 [file Data_Sheet_1.doc]

**Association Between the Weight-adjusted-waist Index and Abdominal Aortic Calcification in US Adults: Results From the NHANES 2013-2014**

Feng Xie,MD^1#^, Yuan Xiao,MD^2#^, Xiaozhong Li,MD^1^, Yanqing Wu, MD, PhD^1*^

**Supplementary Appendix**

**Contents**

**Supplementary tables**

Table S1. Association between BMI level and AAC score in US adults from NHANES 2013-2014.

Table S2. Association between WC level and AAC score in US adults from NHANES 2013-2014.

Table S3. Association of anthropometric indices with AAC score in Pearson correlation analysis.

Table S4. Association between BMI level and severe AAC in US adults from NHANES 2013-2014.

Table S5. Association between WC level and severe AAC in US adults from NHANES 2013-2014.

**Supplementary figures**

Figure S1. ROC curves of each obesity indices.

Table S1. Association between BMI level and AAC score in US adults from NHANES 2013-2014.

| BMI  (kg/m^2^) | N | Crude model  β (95% CI) | Model 1  β (95% CI) | Model 2  β (95% CI) |
| --- | --- | --- | --- | --- |
| Per 1kg/m^2^ increase | 2772 | -0.05 (-0.07, -0.03)* | -0.03 (-0.05, -0.01)* | -0.06 (-0.11, -0.01)* |
| Categorical |  |  |  |  |
| Quintile 1 (14.2-23.8) | 549 | Reference | Reference | Reference |
| Quintile 2 (23.9-26.6) | 560 | 0.34 (-0.04, 0.73) | 0.17 (-0.18, 0.53) | 0.08 (-0.30, 0.47) |
| Quintile 3 (26.7-29.0) | 540 | 0.20 (-0.19, 0.59) | 0.11 (-0.25, 0.48) | -0.07 (-0.52, 0.38) |
| Quintile 4 (29.1-32.7) | 565 | 0.08 (-0.30, 0.46) | 0.09 (-0.26, 0.45) | -0.11 (-0.62, 0.40) |
| Quintile 5 (32.8-51.2) | 558 | -0.65 (-1.04, -0.26)* | -0.52 (-0.88, -0.16)* | -0.84 (-1.51, -0.17)* |
| *P* for trend |  | <0.001 | 0.002 | 0.183 |

* *P* <0.05;

Model 1: adjusted for age, gender and race;

Model 2: adjusted for age, gender, race, WC, education level, alcohol drinking status, eGFR, hemoglobin A1c, serum creatinine, serum uric acid, total cholesterol, triglycerides, total bilirubin, albumin, total 25-hydroxyvitamin D, calcium, phosphorus, hypertension, coronary heart disease, and diabetes mellitus.

Abbreviations: BMI, body mass index; AAC, abdominal aortic calcification;US, the United States; NHANES, National Health and Nutrition Examination Survey; β, effect size; CI, confidence interval.

Table S2. Association between WC level and AAC score in US adults from NHANES 2013-2014.

| Waist circumference  (cm) | N | Crude model  β (95% CI) | Model 1  β (95% CI) | Model 2  β (95% CI) |
| --- | --- | --- | --- | --- |
| Per 10cm increase | 2772 | -0.03 (-0.12, 0.06) | -0.10 (-0.18, -0.01)* | -0.02 (-0.24, 0.19) |
| Categorical |  |  |  |  |
| Quintile 1 (63.5-87.8) | 554 | Reference | Reference | Reference |
| Quintile 2 (87.9-95.4) | 553 | 0.303 (-0.10, 0.70) | 0.11 (-0.26, 0.48) | 0.16 (-0.24, 0.56) |
| Quintile 3 (95.5-102.4) | 548 | 0.61 (0.22, 0.99)* | 0.32 (-0.04, 0.68) | 0.42 (-0.03, 0.86) |
| Quintile 4 (102.5-110.4) | 557 | 0.23 (-0.16, 0.62) | -0.05 (-0.41, 0.32) | 0.08 (-0.44, 0.59) |
| Quintile 5 (110.5-147.0) | 560 | -0.04 (-0.43, 0.34) | -0.33 (-0.69, 0.03) | -0.23 (-0.92, 0.46) |
| *P* for trend |  | 0.591 | 0.016 | 0.721 |

* *P* <0.05;

Model 1: adjusted for age, gender and race;

Model 2: adjusted for age, gender, race, BMI, education level, alcohol drinking status, eGFR, hemoglobin A1c, serum creatinine, serum uric acid, total cholesterol, triglycerides, total bilirubin, albumin, total 25-hydroxyvitamin D, calcium, phosphorus, hypertension, coronary heart disease, and diabetes mellitus.

Abbreviations: WC, waist circumference; AAC, abdominal aortic calcification;US, the United States; NHANES, National Health and Nutrition Examination Survey; β, effect size; CI, confidence interval.

Table S3. Association of anthropometric indices with AAC score in Pearson correlation analysis.

| Variables | r | 95% CI | *P* value |
| --- | --- | --- | --- |
| WWI (cm/√kg) | 0.213 | 0.176, 0.249 | <0.001 |
| BMI (kg/m^2^) | -0.080 | -0.117, -0.042 | <0.001 |
| WC (cm) | -0.011 | -0.048, 0.026 | 0.564 |

Abbreviations: AAC, abdominal aortic calcification; WWI, weight-adjusted-waist index; BMI, body mass index; WC, waist circumference.

Table S4. Association between BMI level and severe AAC in US adults from NHANES 2013-2014.

| BMI  (kg/m^2^) | Severe AAC (n, %) | Crude model  OR (95% CI) | Model 1  OR (95% CI) | Model 2  OR (95% CI) |
| --- | --- | --- | --- | --- |
| Per 1kg/m^2^ increase | 306 (9.64) | 0.95 (0.93, 0.98)* | 0.97 (0.94, 0.99)* | 0.95 (0.89, 1.03) |
| Categorical |  |  |  |  |
| Quintile 1 (14.2-23.8) | 66 (9.64) | Reference | Reference | Reference |
| Quintile 2 (23.9-26.6) | 82 (13.51) | 1.46 (0.96, 2.24) | 1.32 (0.82, 2.14) | 1.19 (0.60, 2.34) |
| Quintile 3 (26.7-29.0) | 69 (11.32) | 1.19 (0.77, 1.86) | 1.21 (0.74, 1.99) | 1.05 (0.51, 2.19) |
| Quintile 4 (29.1-32.7) | 60 (9.12) | 0.94 (0.60, 1.48) | 0.98 (0.60, 1.59) | 0.84 (0.37, 1.94) |
| Quintile 5 (32.8-51.2) | 29 (4.62) | 0.45 (0.26, 0.79)* | 0.55 (0.31, 0.99)* | 0.54 (0.19, 1.57) |
| *P* for trend |  | <0.001 | 0.003 | 0.181 |

* *P* <0.05;

Model 1: adjusted for age, gender and race;

Model 2: adjusted for age, gender, race, WC, education level, alcohol drinking status, eGFR, hemoglobin A1c, serum creatinine, serum uric acid, total cholesterol, triglycerides, total bilirubin, albumin, total 25-hydroxyvitamin D, calcium, phosphorus, hypertension, coronary heart disease, and diabetes mellitus.

Abbreviations: BMI, body mass index; AAC, abdominal aortic calcification;US, the United States; NHANES, National Health and Nutrition Examination Survey; β, effect size; CI, confidence interval.

Table S5. Association between WC level and severe AAC in US adults from NHANES 2013-2014.

| Waist circumference  (cm) | Severe AAC (n, %) | Crude model  OR (95% CI) | Model 1  OR (95% CI) | Model 2  OR (95% CI) |
| --- | --- | --- | --- | --- |
| Per 10cm increase | 306 (9.64) | 0.99 (0.98, 1.01) | 0.99 (0.98, 1.00) | 1.00 (0.97, 1.03) |
| Categorical |  |  |  |  |
| Quintile 1 (63.5-87.8) | 52 (8.08) | Reference | Reference | Reference |
| Quintile 2 (87.9-95.4) | 60 (10.37) | 1.32 (0.81, 2.14) | 1.13 (0.64, 1.97) | 1.11 (0.55, 2.26) |
| Quintile 3 (95.5-102.4) | 91 (13.88) | 1.83 (1.17, 2.87)* | 1.64 (0.99, 2.69) | 1.57 (0.75, 3.28) |
| Quintile 4 (102.5-110.4) | 61 (8.49) | 1.06 (0.66, 1.69) | 0.82 (0.49, 1.39) | 0.84 (0.37, 1.92) |
| Quintile 5 (110.5-147.0) | 42 (7.38) | 0.91 (0.54, 1.53) | 0.79 (0.44, 1.41) | 0.89 (0.30, 2.67) |
| *P* for trend |  | 0.375 | 0.028 | 0.756 |

* *P* <0.05;

Model 1: adjusted for age, gender and race;

Model 2: adjusted for age, gender, race, BMI, education level, alcohol drinking status, eGFR, hemoglobin A1c, serum creatinine, serum uric acid, total cholesterol, triglycerides, total bilirubin, albumin, total 25-hydroxyvitamin D, calcium, phosphorus, hypertension, coronary heart disease, and diabetes mellitus.

Abbreviations: WC, waist circumference; AAC, abdominal aortic calcification;US, the United States; NHANES, National Health and Nutrition Examination Survey; β, effect size; CI, confidence interval.


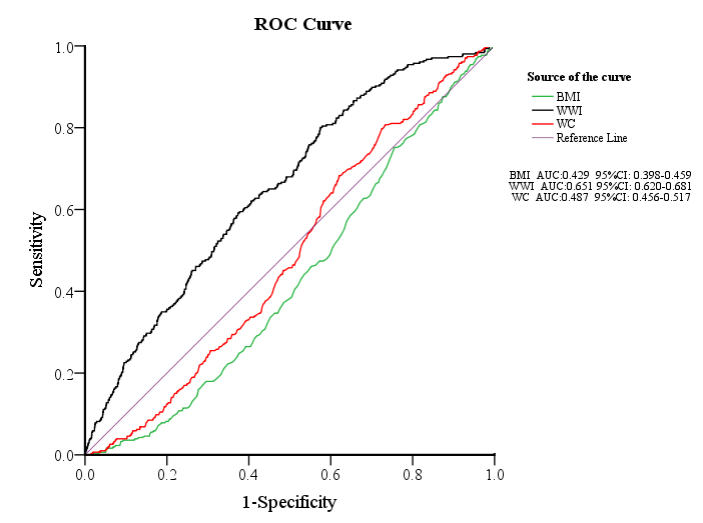


Figure S1. ROC curves of each obesity indices. BMI, body mass index; WWI, weight-adjusted-waist index; WC, waist circumference; ROC, receiver operating characteristic; AUC, area under the curve.
